# An interventional exploratory study to assess the effect of footwear on postural stability and strategy during quiet standing

**DOI:** 10.1080/23335432.2021.1985610

**Published:** 2021-10-13

**Authors:** J. Hausselle, A. G. Haddox, J. Kasitz, A. Azoug

**Affiliations:** School of Mechanical & Aerospace Engineering, Oklahoma State University, Stillwater, USA

**Keywords:** Footwear, stability, postural sway, postural strategy

## Abstract

A stable quiet stance is achieved by controlling the relative position of the center of pressure and the vertical projection of the center of mass. The best postural performances include efficient strategies to mitigate external perturbations. Footwear impacts postural stability and strategy by affecting cutaneous proprioception and ankle proprioception in the case of heeled shoes. The purpose of this study was to quantify the effects of four common footwear conditions, *i.e*. barefoot, sports, flats, and heels, on postural stability and strategy during quiet standing of healthy young women. Postural stability and strategy were assessed overall and in the antero-posterior and medio-lateral directions using five parameters: total sway, average center of pressure (COP) velocity, α value computed using detrended fluctuation analysis, hip over ankle ranges of motion, and power of the COP time series. Significant differences with barefoot were consistently found when wearing heels, namely a decrease in postural sway and average COP velocity. Results seemed counter-intuitive as they indicate an apparent increase in postural stability when wearing heels. A deeper analysis revealed a more complex scheme. A potential tightening of the motion when wearing heels, combined with an increase of the neutral plantarflexion angle, shifts the postural strategy towards a predominant hip strategy. Finally, proprioception did not play a key role. This study highlighted the complexity of the multifactorial interactions between footwear characteristics and postural strategies. Additional work is needed to develop footwear that will enhance postural stability of populations at risk, such as pregnant women or the elderly.

## Introduction

Postural stability is the ability to maintain an erect position and is necessary to perform daily tasks without falling (Sousa et al. 2012; Mika et al. 2016). A stable quiet stance is achieved by minimizing the distance between the location of the center of pressure (COP) under the feet and the vertical projection of the center of mass (COM) of the body on the ground. The overarching goal is to maintain the projection of the COM within the base of support (Sousa et al. 2012).

The best postural performances are energy efficient since they rely on the skeletal and ligamentous systems to minimize muscle work and thus fatigue (Hastings et al. 2018). In addition, a good posture includes efficient strategies to mitigate external perturbations and allows for a fast recovery following a potential loss of balance. Impaired postural control can be due to joint laxity or issues dealing with incoming sensory information (Liu et al. 2012). In any case, lower back and limb muscle tones must increase to restore postural stability. This increase, in turn, leads to muscle fatigue and raised joint contact forces. The two main strategies to maintain a stable posture utilize the ankle and hip joints. The ankle strategy is defined by an in-phase sagittal rotation of the leg and trunk segments, *i.e*. the ankle and hip joint rotates in the same direction, whereas the hip strategy is defined by an anti-phase rotation (Creath et al. 2005). Efficient postural strategies are typically a mix of these two strategies, with the ankle strategy dominating at low sway frequencies, *i.e*. below 1 Hz, and the hip strategy dominating at higher frequencies, up to 5 Hz (Creath et al. 2005; Saffer et al. 2008; Hay and Wachowiak 2017).

Footwear can affect the optimal control mechanisms in several ways (Federolf et al. 2012). The simple fact of wearing a shoe may increase cutaneous proprioception, mostly due to the mechanoreceptors found in the plantar surface of the feet (Aboutorabi et al. 2016; Alghadir et al. 2018; Li et al. 2019). Cutaneous proprioception is one of the most important sensory systems participating to maintaining postural stability (Lord et al. 1991). In addition, regardless of the predominant postural strategy adopted, ankle proprioception is a critical part of the feedback loop established by the central nervous system to maintain a stable erect posture during quiet standing. By modifying the neutral ankle joint angle, heeled shoes influence this proprioceptive process and may thus modify the postural strategy. Mika et al. (2016) showed that even low-heeled shoes (40 mm) lead to abnormal COP excursions in both the antero-posterior (AP) and medio-lateral (ML) directions. The instability increases in the case of high-heeled shoes (Mika et al. 2016; Emmanouil and Rousanoglou 2018). Finally, the strain in the arch of the foot provides additional proprioceptive feedback (Kogler et al. 1996), thus the shape of the shoe insole and its stiffness may also affect the postural performance.

Numerous parameters can characterize postural performance (Yamamoto et al. 2015). Traditional parameters are based on the motion and velocity of the COP, usually decomposed along the AP and ML directions. However, interpretations of these parameters differ. Although high postural sway, computed as the total length of the COP path, is often correlated with low postural stability (Paillard and Noe 2015), reduced sway and tightening of the motion may also constitute a mechanism to handle fear of falling (Adkin et al. 2002). Average COP velocity has been proposed as a better indicator of postural stability than COP displacement since joint velocities are directly used by the body as postural feedback (Masani et al. 2003). Non-linear approaches, such as fractal analyses, can highlight subtle changes in the postural strategy that are not detected by the traditional linear analyses of sway and COP velocity (Doyle et al. 2004; Noda and Demura 2006). Notably, these non-linear analyses can assess the adaptability of a system, *i.e*. its capability to efficiently react to external perturbations. Nonetheless, they have been scarcely applied to quantify the effects of footwear on postural control.

Footwear plays a critical part in our lives since our main daily activities include standing (about 80 minutes/day) and walking (about 180 minutes/day) (Johansson et al. 2019). Improper footwear can lead to an increased risk of falling, especially in the elderly (Frey and Kubasak 1998; Brenton-Rule et al. 2011) but can also lead to a variety of occupational musculoskeletal disorders (Halim et al. 2012; Anderson et al. 2016). Several studies have quantified the effects of heeled-shoes (Mika et al. 2016; Emmanouil and Rousanoglou 2018), occupational footwear (Chander et al. 2014), sandals (Alghadir et al. 2018), minimalist shoes (Cudejko et al. 2020), as well as sport and so-called unstable shoes (Landry et al. 2010; Federolf et al. 2012) on postural stability. Most studies have focused on elderly patients and results regarding postural stability are inconsistent (Federolf et al. 2012). In addition, there exists a dearth of information regarding the effects of footwear on postural strategy including proprioception.

The purpose of this study was to quantify in healthy young women the effect of three commercially available footwear, *i.e*. sports, flats, and heels, on postural stability and strategy during quiet standing. This new knowledge will shed light on possible causes of footwear-related musculoskeletal disorders in working women and pave the way for corrective or preventive interventions. We hypothesized that footwear will affect postural performance. Specifically, we expected that postural stability would be reduced when increasing ankle plantarflexion, and that the hip strategy would become predominant. An increase in ankle plantarflexion is expected to reduce the efficacy of the plantar flexor muscles in controlling the sway (Emmanouil and Rousanoglou 2018), thus leading to a postural control that would rely more on the hip muscles. Furthermore, we expected proprioceptive effects to increase when wearing shoes with stiffer insoles such as flats and heels. Proprioception plays a critical part in postural control and is in part provided by mechanoreceptors found in the sole of the foot. Thus, a stiffer insole may enhance plantar proprioception and thus postural feedback (Brenton-Rule et al. 2011).

## Materials and Methods

This study is a preliminary interventional study realized on ten healthy subjects.

### Setting

This study was conducted under the approval of the University’s Institutional Review Board and all subjects signed an informed consent form. All data collection took place in the BAMM Lab located on the Oklahoma State University campus in Stillwater, Ok, USA. Subjects were recruited and participated in data collection between February and September 2019.

### Subjects

Subjects were recruited among college students at Oklahoma State University via flyers posted on campus and were screened based on four exclusion criteria: diagnosed osteoarthritis, history of lower limb surgeries, existing cardiac conditions, and/or diabetes. Ten healthy females between the ages of 18 and 30 and with body mass index (BMI) below 26 were included (age: 22.8 ± 4.4 years old, BMI: 22.4 ± 2.4 kg/m^2^).

### Footwear

**Figure 1.**
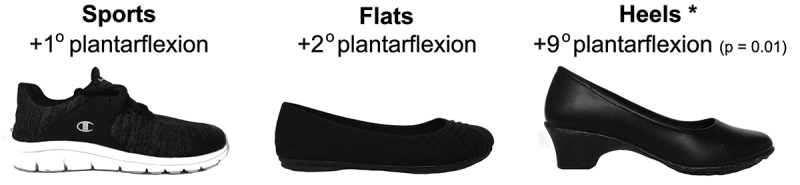
Types of footwear studied, in addition to barefoot, with average plantarflexion angle difference with the barefoot condition. An asterisk signals a significant difference (p < 0.05). Only when wearing heels did the subjects exhibit a significant increase in plantarflexion compared to barefoot

**Figure 2.**
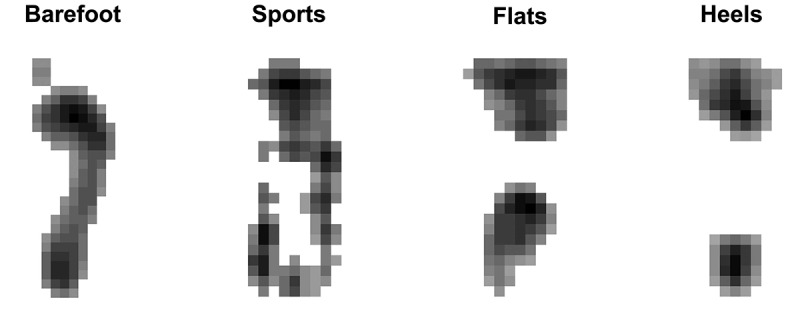
Examples of pressure maps for each footwear condition for a single subject

Four footwear conditions were tested: barefoot, sport shoes (Gusto Runner Champion), flat shoes (Bree), and 45 mm-high heels (Tressa Pump, base: 37 mm in length and 31 mm in width for a size 8) (Figure 1). Shoe size was determined based on each subject’s experience, and then put on to check for proper fit based on the subject’s feedback. The total contact surface area depends on footwear condition, shoe size, and subject posture (Figure 2).

### Data collection protocol

**Figure 3.**
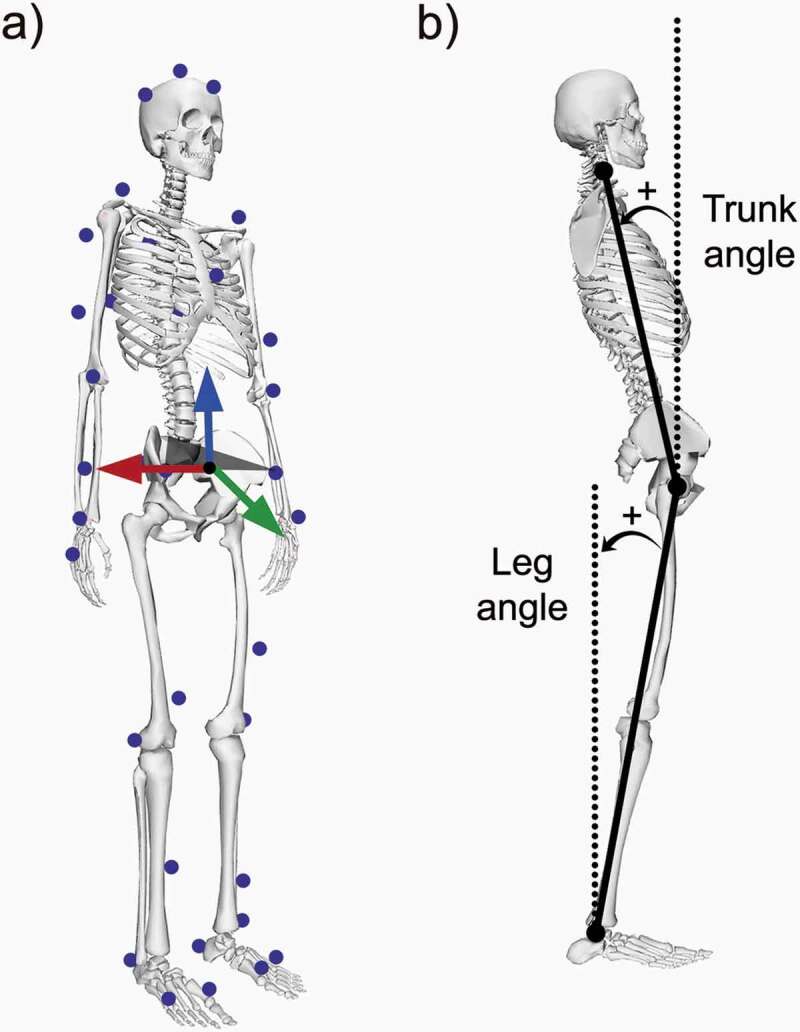
a) Motion analysis markerset (blue) and pelvis coordinate system (X axis in red, Y in green, and Z in blue). b) Trunk and leg angle definitions

**Figure 4.**
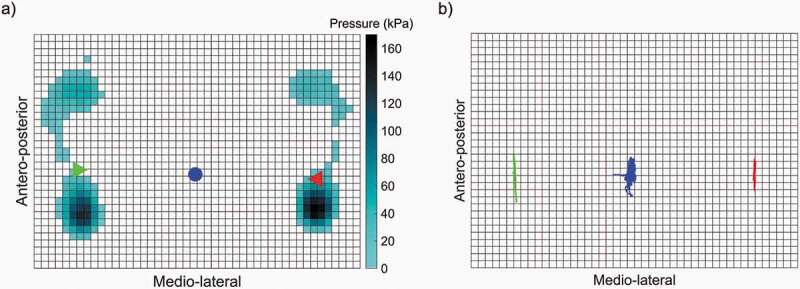
a) Example of instantaneous pressure map during quiet standing barefoot (pixel = 8.5 × 8.5 mm) with COP under the left foot (green triangle), under the right foot (red triangle), and global (blue circle). b) Example of COP displacement during quiet standing barefoot. The COP of interest is the global one (blue)

Subjects were instrumented with 37 motion analysis markers (Figure 3a) following the standard Rizzoli full-body protocol implemented in the software (Motive, Natural Point Corvallis, OR). The Rizzoli body protocol combines the Rizzoli lower body protocol (Leardini et al. 2007) and the Rizzoli trunk protocol (Leardini et al. 2011) to create a full set of markers. Subjects were then asked to stand erect and look straight to the wall facing forward in their most natural position for 300 s on an instrumented treadmill at zero speed (Noraxon USA Inc., Scottsdale, AZ). Plantar pressure distributions were recorded at 100 Hz. Each pressure sensor embedded in the treadmill belt measured 8.5 x 8.5 mm (Figure 4). Kinematics data were recorded at 120 Hz using an eight-camera motion analysis system (NaturalPoint, Inc., Corvallis, OR).

Standing barefoot was the first condition tested, whereas the order of the other conditions was randomized for each subject. Each footwear condition was tested once.

We estimated the effect of footwear on the neutral ankle plantarflexion by computing the average angle between the horizontal line and the segment connecting the markers on the ankle and the head of the first metatarsal. The increase in ankle plantarflexion was defined for each footwear condition as the difference between the plantarflexion of the condition of interest and the plantarflexion of the barefoot condition (Figure 1).

### Data processing

Data processing was performed using MATLAB R2019a (The MathWorks, Inc., Natick, MA, USA). Motion analysis data was downsampled to 100 Hz to match the pressure acquisition frequency. For each trial, the first ten seconds were discarded, and the next sixty seconds were used for analysis, similarly to previous studies (Chander et al. 2014; Mika et al. 2016). The positions of the hip joint centers were estimated using the Hayes’ pelvis model (Davis et al. 1991). To minimize any misalignment between the subject and the treadmill, pressure maps (and marker coordinates) were expressed in the pelvic coordinate system at each instant in time (Figure 3a). The instantaneous COP locations in the AP and ML directions were computed using a weighted average of the pressure map (Figure 4).

### Outcome measures

#### Postural stability

Global postural stability was assessed by computing three parameters: total sway, average COP velocity, and COP velocity variability.

The total sway was defined as the total distance traveled by the COP during the trial (Paillard and Noe 2015). A smaller value often characterizes a higher postural stability but results from an increase in rigidity due to the fear of falling.

The instantaneous COP velocity was computed as the first derivative of the COP time series using the central five-point finite difference method, and then averaged over the trial to obtain the average COP velocity. Following the recommendation of Giovanini et al. (2017) and before computing the velocity variability, the COP time series were downsampled to 25 Hz and low-pass filtered using a second-order Butterworth filter with a cut-off frequency of 10 Hz. A higher value characterizes a higher postural stability since reactions to external perturbations should be faster.

The COP velocity variability was determined using detrended fluctuation analysis (DFA) (Hausdorff et al. 1995). The output of the DFA is the scaling exponent α, which quantifies the long-range correlations of a time series. We also performed surrogate data analysis by computing α values for each randomly shuffled time series, to ensure that the use of DFA was appropriate. A higher value characterizes a more adaptable system that can efficiently respond to external perturbations (Hausdorff 2007; Federolf et al. 2012).

Similarly, to global postural stability, directional stability in the AP and ML directions was assessed using the same three parameters.

#### Postural strategy

Postural strategy was assessed by two parameters: the ratio of the ranges of motion (ROM) of the hip and ankle joints and the frequency-related power distribution of the COP time series (Paillard and Noe 2015).

The sagittal plane was defined using the antero-posterior and vertical axes of the pelvis coordinate system previously computed (Davis et al. 1991). To determine the ROM ratio, three body landmarks were projected on the sagittal plane at each instant in time: the marker on the acromion process, the virtual hip joint center, and the marker on the lateral malleolus. For each pair of projected landmarks (right and left), the average point was computed and then used to define the two segments of interest (Figure 3b): the trunk segment connecting the hip joint center to the acromion process and the leg segment connecting the lateral malleolus to the hip joint center. The hip angle was computed as the angle between the trunk segment and the vertical line passing through the hip joint center, whereas the ankle angle was computed as the angle between the leg segment and the vertical line passing through the lateral malleolus. The ROM was then computed for each joint as the difference between the maximum and minimum angle over the entire trial. Finally, the ROM ratio was defined as the ratio between the hip and ankle ROMs. A ratio of 1 indicates equal influence of the hip and ankle joints, whereas a higher ratio indicates a predominant hip strategy.

Power of the COP time series was computed as the area under the power spectral density curve obtained via Fast Fourier Transform. Finally, to characterize power distribution, we computed the percentage of power contained in three frequency bands: below 0.5 Hz, which represents visuovestibular control, between 0.5 and 2.0 Hz, which represents cerebellar control, and above 2.0 Hz, which represents proprioceptive control (Paillard and Noe 2015). A higher value in a band characterizes a predominant corresponding control strategy.

### Statistical analysis

Statistical analyses were performed using SPSS 26 (IBM, Armonk, NY, USA). For each parameter characterizing postural stability or postural strategy, the effect of the footwear condition was first determined using repeated measures analysis of variance (ANOVA). The Mauchly’s test was performed to ensure that the assumption of sphericity was valid, which is necessary for running repeated measures ANOVA. In case of violation, we applied the Greenhouse-Geisser correction. Finally, if a significant effect was found, post hoc analyses were performed using multiple pairwise comparisons with Bonferroni correction (Jaccard et al. 1984). The p value was set at 0.05.

## Results

### Postural stability

**Figure 5.**
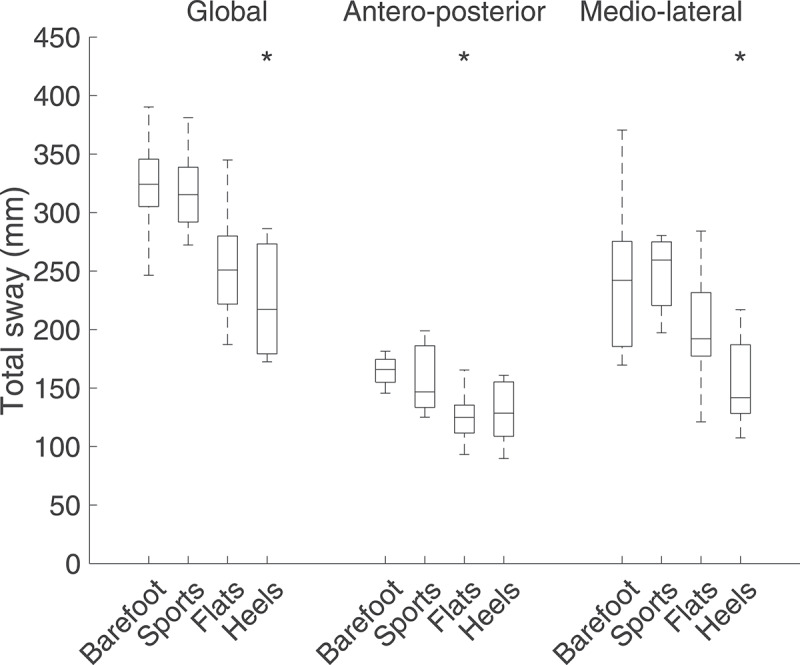
Total sway over 60 s for the four footwear conditions. For each boxplot, the central line represents the median value, the bottom and top of the box represent the 25^th^ and 75^th^ percentiles, and the whiskers are linked to the most extreme data points not considered outliers. An asterisk signals a significant difference (p < 0.05) when compared to the barefoot condition. The global postural sway was significantly reduced when wearing heels (p = 0.01)

Total postural sway decreased throughout the footwear conditions (from barefoot to sports, flats, and heels), but was only significantly lower than the barefoot condition when wearing heels (p = 0.01) (Figure 5). When decomposing the sway in the AP and ML directions, the same trend was observed, but the sway was only significantly lower in the AP direction when wearing flats (p = 0.03), and in the ML direction when wearing heels (p = 0.01). Regardless of the footwear condition, the ML sway was significantly higher than the AP sway (p < 0.02).

**Figure 6.**
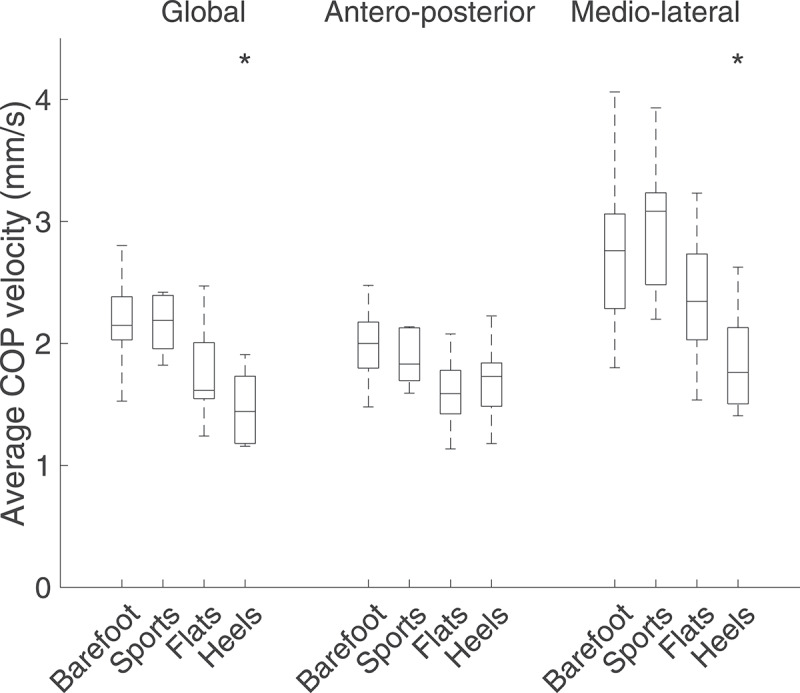
Average COP velocities over 60 s for the four footwear conditions. An asterisk signals a significant difference (p < 0.05) when compared to the barefoot condition. The global average velocity was significantly reduced when wearing heels (p = 0.011)

Global average COP velocity decreased throughout the footwear conditions (from barefoot to sports, flats, and heels), but was only significantly lower than the barefoot condition when wearing heels (p = 0.011) (Figure 6). When decomposing the velocity in the AP and ML directions, the average COP velocity was significantly reduced in the ML direction when wearing heels (p = 0.017).

**Figure 7.**
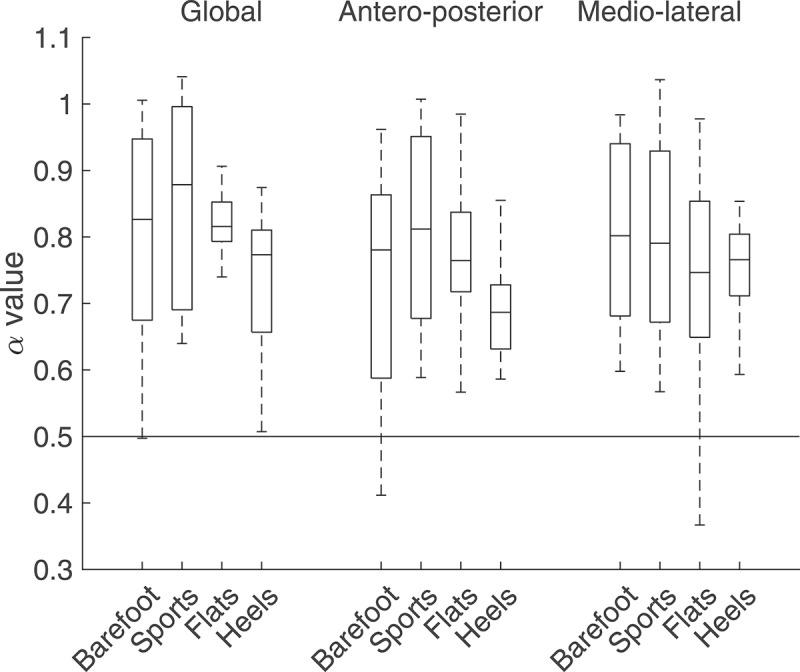
α values computed using detrended fluctuation analysis for the four footwear conditions. A value of 0.5 indicates a random signal, whereas higher values indicates a more complex signal corresponding to a more adaptable system. Although there was a decrease of the global and AP α values when wearing heels, it was not significant

Footwear condition did not have any significant effect on the COP velocity variability. Although the global and AP α values decreased when wearing heels, the decrease was not significant. The average α values varied between 0.70 and 0.85 (Figure 7), which indicates a fairly adaptable system capable of handling external perturbations (Hausdorff 2007; Federolf et al. 2012). For the surrogate series, the average α values in the global, AP, and ML directions were 0.49 (± 0.09), 0.46 (±0.08), and 0.48 (± 0.09), respectively. For each direction and each footwear condition, these values were close to the theoretical value for a random signal, *i.e*. 0.5, and significantly lower (p < 0.05) than the ones obtained for the initial time series, confirming the validity of the DFA.

### Postural strategy

**Figure 8.**
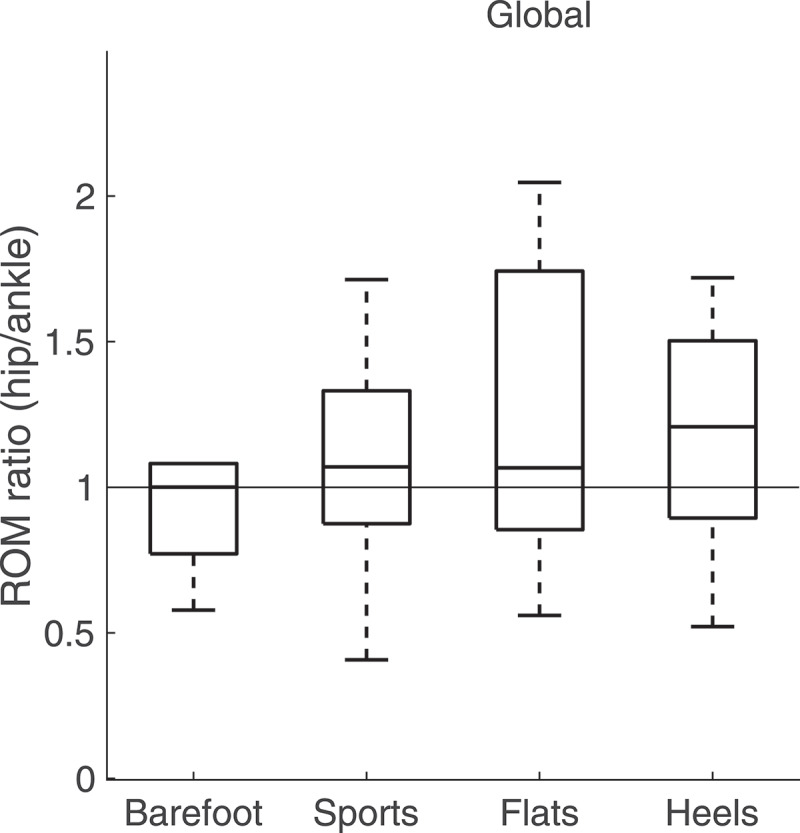
Range of motion ratios between the trunk and leg angles over 60 s for the four footwear conditions. Although there was an increase in the use of the hip joint relative to the ankle joint between each footwear condition and the barefoot condition, it was not significant

Footwear condition did not have any significant effect on the ROM ratio between the hip and ankle (Figure 8). Although there was a trend towards an increased use of the hip joint for all footwear conditions in comparison to the barefoot condition, this trend was not significant.

**Figure 9.**
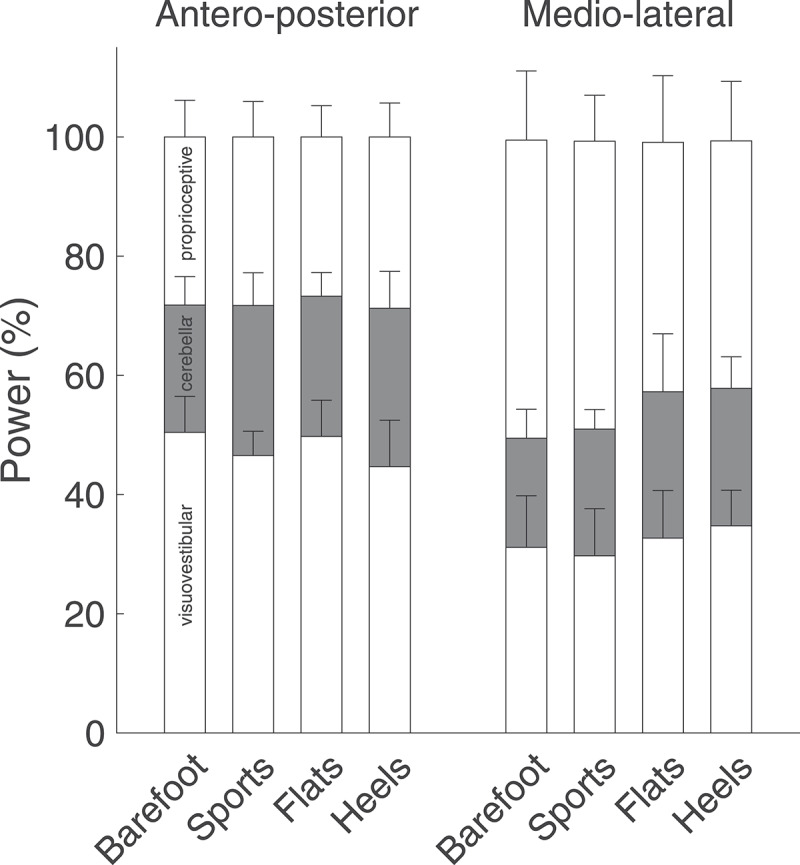
Percentage of total power contained in three frequency bands: below 0.5 Hz (visuovestibular control), between 0.5 and 2.0 Hz (cerebellar control), and above 2.0 Hz (proprioceptive control). The power developed by the proprioceptive system was significantly higher in the ML direction than in the AP direction for all the footwear conditions (p < 0.01)

Similarly, footwear condition did not have any significant effect on the relative distribution of power in the three frequency bands (Figure 9). However, the power developed by the proprioceptive system was significantly higher in the ML direction than in the AP direction for all the footwear conditions (p < 0.01).

## Discussion

Our goal was to quantify the effects of four different types of footwear on postural stability and strategy during quiet standing of healthy young women. The overall finding is that, except for heeled shoes, footwear conditions do not fundamentally alter postural stability or strategy in young healthy subjects. Our hypothesis was only partially validated since significant differences with respect to standing barefoot were only consistently found when wearing heels. Wearing sport shoes was the condition that was the closest to barefoot since we did not find significant differences for any of the parameters studied. The shoes used did not enhance proprioception in young and healthy subjects. Our findings highlight the complexity of the multifactorial interactions between footwear characteristics and postural strategies, and the difficulty of grasping the effects of footwear on postural performance.

### Postural stability

#### Heels

Global sway decreases when wearing heels compared to standing barefoot, unexpectedly indicating a theoretically higher stability (Paillard and Noe 2015).

As this behavior is mostly due to ML motions, we did not observe the increased AP sway previously reported (Mika et al. 2016; Emmanouil and Rousanoglou 2018). Emmanouil and Rousanoglou (2018) showed that wearing 110 mm-high heels increased AP and ML sway when compared to barefoot. However, wearing 65 mm-high heels only increased sway in the AP direction. The main differences between the two protocols are the heel height and base size. Emmanouil and Rousanoglou (2018) used 65 mm- and 110 mm-high heels with a base of 10 mm x 10 mm, whereas we used 45 mm-high heels with a base of 40 mm (length) x 35 mm (width). We used heels with a wider base since our goal was not to minimize the base of support to create imbalances, but to modify the neutral ankle joint angle, and thus potentially affect the ankle proprioception.

Similarly, Mika et al. (2016) compared postural sway between barefoot, 40 mm-high heels, and 100 mm-high heels, and found significant increases in both the AP and ML directions between the heels and barefoot conditions. Again, the main difference with our study is the type of heel tested. Mika et al. (2016) used a stiletto-heeled shoe, with a base of 100 mm^2^, compared to our base of 1,400 mm^2^. Discrepancy between these studies indicate that the base area under the heel plays a critical role in postural stability. To verify this hypothesis, future studies should focus on testing heeled shoes of similar height but with different heel support areas.

Although our findings may seem counter-intuitive, reduced sway can also be interpreted as a tightening of the motion in response to a fear of falling (Adkin et al. 2002). Unfortunately, we did not ask our subjects if they were used to wearing heels but, if it was not the case, an increased fear of falling may explain these results. The effect of the fear of falling on postural stability could be assessed in a future study comparing frequent and occasional heel wearers. In addition, subjects were instructed to keep their eyes opened, which may have skewed the results since visual feedback is believed to play a more important role in postural control than proprioception, although less for younger subjects than older ones (Doyle et al. 2004). Replicating these tests with eyes closed would assess the effect of visual information on postural stability.

Characterizing postural stability based solely on COP locations has been previously criticized (Doyle et al. 2004) but Masani et al. (2003) argued that COP velocity is a more accurate assessment of postural control since it is linked to postural feedback. The average COP velocities followed the same trend as the total sway. The COP velocity is significantly lower when wearing heels than when standing barefoot, again mostly due to the behavior of the system in the ML direction.

The COP velocity variability exhibits a relatively high value independently of the footwear conditions, meaning that the postural system remained adaptable, *i.e*. capable of efficiently responding to external perturbations (Doyle et al. 2004; Federolf et al. 2012; van Emmerik et al. 2016). The coefficient α decreases, although not significantly, between barefoot and heels conditions. This trend confirms the tightening of the motion previously mentioned to explain the decrease in total sway. Tightening is obtained through increased muscular contractions, notably of the plantarflexors, which in turn may inhibit the ability of the system to quickly react to perturbations.

#### Sport shoes

We found that wearing sport shoes did not significantly alter sway and, hence, postural stability (Paillard and Noe 2015). While wearing ‘standard shoes’ similarly did not modify balance scores in one study (Alghadir et al. 2018), the AP sway significantly differed between barefoot and two types of sport shoes in another (Brenton-Rule et al. 2011). However, subjects in Brenton-Rule et al. (2011) were older (age = 74 ± 5 years old) and were asked to focus their sight on a white spot positioned at their eye level. Since we focused on young healthy subjects, we safely assume that they did not exhibit age-related sensory decline and thus that the enhanced cutaneous proprioception provided by the sport shoes may not be as significant as for older subjects.

The global α value tends to increase between barefoot and sport shoes conditions. Federolf et al. (2012) performed DFA on the principal components of the time series combining the three-dimensional positions of 28 motion analysis markers and showed a significant decrease of α for the first principal component when wearing an athletic shoe in comparison to standing barefooted. This approach is radically different from the one presented in this paper so comparing the α values and trends is not recommended, but conclusions are similar, *i.e*. sport shoes tend to increase the adaptability of the postural system.

#### Flats

We found a significantly higher AP stability when wearing flats in comparison to standing barefooted. This result was expected since the relatively stiff insole of these shoes increases cutaneous proprioception, as observed in older subjects (Aboutorabi et al. 2016).

### Postural strategy

For sport shoes and flats conditions, the optimal postural strategy adopted by the body to maintain a quiet upright position remained fundamentally similar.

Regardless of the footwear condition, the total sway remains higher in the ML direction than in the AP direction, as previously reported (Winter and Eng 1995; Yodchaisarn et al. 2018). Winter and Eng (1995) established that different postural strategies are responsible for controlling sway in these two directions: the AP sway is regulated by the ankle dorsi/plantarflexion, hence related to an ankle strategy, whereas the ML sway is regulated by the hip abduction/adduction, hence related to a hip strategy. We found that the ratio ML sway/AP sway was higher for the heels condition when compared to any other condition, which highlights that the postural system tends to promote the hip strategy when wearing heels. Consequently, we observed a possible increase of the reliance on the hip strategy when wearing heels compared to barefoot.

When wearing heels, the motion tightens through an increased use of the plantarflexors, the total sway decreases, and additional control may be provided using the hip muscles to relieve the already overloaded ankle muscles. Furthermore, the plantarflexed neutral position adopted when wearing heels may alter ankle muscle activations, forcing the body to rely on hip muscles since there only exists a handful of muscle synergies ensuring postural balance (Torres-Oviedo and Ting 2007).

Another critical aspect of postural control to consider when comparing different footwear is proprioception. Shoes exhibit several characteristics that can affect cutaneous proprioception, including insole shape and stiffness, and collar height and stiffness (Lord et al. 1991; Mika et al. 2016; Aboutorabi et al. 2016; Emmanouil and Rousanoglou 2018; Li et al. 2019; Cudejko et al. 2020). To assess potential proprioceptive effects of the shoes tested in this study, we computed the percentage of power of the COP time series in three frequency band, with the band above 2 Hz representing proprioceptive effects. We did not find a significant effect of footwear, showing that changes observed in postural stability and strategy were not driven by an enhanced or degraded proprioception. One of our initial hypotheses that proprioceptive effects would increase when wearing stiffer shoes such as flats and heels was thus not validated.

### Limitations and future work

This study has several limitations. First, the limited number of subjects may have hidden significant differences or resulted in false positive influences in the ANOVA. The repeated measure ANOVA relies on the assumption of normality, which is violated here. Indeed, assessing a normal distribution on a low number of data points is almost impossible. However, the repeated measure ANOVA is relatively robust to this violation (Glass et al. 1972; Lix et al. 1996), meaning that, although the accuracy may be slightly lower, results are not considered erroneous. Second, we only tested young healthy female subjects, so extrapolation of our results to older or male subjects is not recommended. Third, we did not ask our subjects if they were used to wearing heels, which would impact their fear of falling. Finally, we did not measure the AP and ML ground reaction forces, which could be useful to characterize postural strategy (Hay and Wachowiak 2017). Future studies will focus on including additional female subjects as well as male subjects. Given our findings regarding the potential importance of the heel base, we will also focus on assessing the relative effects of the heel area and height on postural stability and muscle synergies.

## Conclusion

Our goal was to quantify the effects of four common footwear conditions, *i.e*. barefoot, sports, flats, and heels, on postural stability and strategy during quiet standing of healthy young women. Our hypothesis that footwear condition affects more than one aspect of the postural performance was only partially validated. Significant differences with respect to standing barefoot were consistently found when wearing heels, which highlighted a potential tightening of the motion, probably due to a fear of falling. The limited effect of footwear on both postural stability and strategy in these young healthy subjects provides a baseline for studies evaluating the influence of other factors on the fear of falling, such as the change in weight distribution due to obesity or pregnancy.
